# miR-1185-1 and miR-548q Are Biomarkers of Response to Weight Loss and Regulate the Expression of *GSK3B*

**DOI:** 10.3390/cells8121548

**Published:** 2019-11-30

**Authors:** Marcos Garcia-Lacarte, Maria L. Mansego, M. Angeles Zulet, J. Alfredo Martinez, Fermin I. Milagro

**Affiliations:** 1 Department of Nutrition, Food Science and Physiology, University of Navarra, 31008 Pamplona, Spain; mglacarte@unav.es (M.G.-L.); mlmansego@making-genetics.eu (M.L.M.); mazulet@unav.es (M.A.Z.); jalfmtz@unav.es (J.A.M.); 2 Centre for Nutrition Research, University of Navarra, 31008 Pamplona, Spain; 3 CIBERobn, Centro de Investigación Biomédica en Red de la Fisiopatología de la Obesidad y Nutrición, ISCIII 28029 Madrid, Spain; 4 IdiSNA, Navarra’s Health Research Institute, 31008 Pamplona, Spain; 5 Madrid Institute of Advance Studies (IMDEA), IMDEA Food, 28049 Madrid, Spain

**Keywords:** microRNA, biomarker, miRNA-Seq, inflammation, obesity

## Abstract

The aim of the present investigation was to identify putative miRNAs involved in the response to weight loss. Reverse-transcribed RNA isolated from white blood cells (WBCs) of a subpopulation from the Reduction of the Metabolic Syndrome in Navarra-Spain (RESMENA-S) study (low-responders (LR) and high-responders (HR)) was hybridized in a gene expression microarray. Moreover, miRNAs were sequenced by miRNA-Seq. It was found that miR-548q and miR-1185-1 were overexpressed in HR, both in the microarray and in the miRNA-Seq. A bioinformatic prediction of putative target genes of the selected miRNAs found that *GSK3B*, a putative target for miR-548q and miR-1185-1, was downregulated in HR. Particular 3′-UTR binding regions of *GSK3B* were cloned downstream of the firefly luciferase gene. HEK-293T cells were co-transfected with either 0.25 μg of empty pmiR-GLO or pmiR-GLO-548q-3′-UTR/pmiR-GLO-1185-1-3′-UTR, and 7.5 pmol of miR-548q/miR-1185-1 mimics, demonstrating that miR-1185-1 bound to the 3′-UTR region of *GSK3B*. THP-1 cells were transfected with either 20/40 nM of miR-548q/miR-1185-1 mimics, evidencing that miR-1185-1inhibited the expression of the gene when transfected at doses of 20/40 nM, whereas miR-548q inhibited *GSK3B* expression at a dose of 40 nM. As a conclusion, miR-548q and miR-1185-1 levels in WBCs are biomarkers of response to weight-loss diets and could be involved in the regulation of the proinflammatory gene *GSK3B*.

## 1. Introduction

The prevalence of obesity is increasing worldwide, as well as their accompanying comorbidities, such as cardiovascular disease, type 2 diabetes, chronic kidney disease, nonalcoholic fatty liver disease, and musculoskeletal disorders [1]. Obesity, insulin resistance and type 2 diabetes are closely related to a chronic inflammatory state. White adipose tissue (WAT) in obese individuals presents a higher infiltration of macrophages and an increased secretion of proinflammatory cytokines than non-obese WAT, leading to greater macrophage recruitment that aggravates the inflammatory response [2]. However, obesity status is complex and multifactorial, and lifestyle modifications including dietary-induced weight loss and physical activity are not equally effective for every person. Therefore, continuous efforts are being made to develop personalized approaches based on nutrigenetic and nutrigenomic data [3]. Consequently, novel biomarkers of prognosis or even of response to dietary treatments to overcome obesity and its inflammatory state are urgently required.

In this context, microRNAs (miRNAs) are small single stranded noncoding RNA molecules, approximately 18–25 nucleotides in length, that bind to the 3′-UTR region of target genes, regulating their transcription and usually resulting in the degradation of mRNA or inhibition of the translation [4]. A single miRNA has different target genes, and one single gene transcript is regulated by several miRNAs, generating an enormous cluster of miRNA-target gene regulatory pathways [5].

The enrolment of miRNAs in gene regulation highlights their impact on the control of metabolic homeostasis and their implication in the development of obesity- and metabolic syndrome-related comorbidities [6,7]. For example, miRNAs are involved in the control of adipogenesis, browning of adipose tissue or inflammation [8,9,10], and have the capacity to modulate glucose and lipid metabolism in the liver [11], glucose-stimulated insulin secretion [12], or leptin signaling in the hypothalamus [13]. On the other hand, there are several examples in which miRNAs are used as biomarkers or clinical tools for diagnosis and prognosis of several diseases, including obesity and diabetes [14,15,16,17].

One of the areas where miRNA biomarkers could be helpful is in the prediction of the response to a weight loss intervention [18]. Many miRNAs have been proposed as predictors of the response to dietary interventions. For example, circulating miR-935 and miR-140 levels were described as biomarkers of the magnitude of weight loss in an exercise and nutritional intervention [19]. Moreover, our group has previously demonstrated that miRNAs expression in blood cells could be also used as a prognostic biomarker of weight loss [20,21]. In this study, we aimed to search for miRNA-type biomarkers capable of predicting the response to a specific dietary treatment, in an attempt to elucidate their impact on the expression of target genes and their mechanism of action.

## 2. Experimental Section

A subsample from the RESMENA (Metabolic Syndrome Reduction in Navarra) nutritional intervention trial was used in the present study. 96 metabolic syndrome adults underwent two hypocaloric diet (−30% of energy restriction) interventions. As no differences were found either in anthropometric or in biochemical variables between groups after the intervention, both dietary groups were merged to increase the statistical power of the study. After the dietary intervention, aiming to identify miRNA-type biomarkers implicated in the response to the weight-loss intervention, participants were categorized into “high responders” (HR), when weight loss was ≥8%, and “low responders” (LR) when weight loss was ≤8%, as previously published [21]. After categorization, samples from both groups were randomly selected and were adjusted for sex, gender and weight prior to microarray analysis.

Accordingly, miRNA-Seq samples were selected randomly and adjusted for sex, gender and weight.

Plasma concentrations of selected adipokines were analyzed with enzyme-linked immunosorbent assay kits (ELISA) and measured by an autoanalyzer system (Triturus, Grifols SA, Barcelona, Spain), following the manufacturer’s instructions.

The study was performed following the CONSORT 2010 guidelines and received approval from the Ethics Committee of the University of Navarra (065/2009) and was registered at www.clinicaltrials.gov (NTC01087086). All participants provided written informed consent for participation.

### 2.1. RNA Isolation and Reverse Transcription

Plasma, erythrocytes and white blood cells (WBCs) were separated from whole blood by centrifugation at 1100 g at 4 °C for 15 min (Model 5804R, Eppendorf AG, Hamburg, Germany), and were stored at −80 °C until analyses. Total RNA from WBCs was extracted using TRIzol reagent (Life Technologies, Carlsbad, CA, USA) according to the manufacturer’s protocol. cDNA was synthesized using 0.5 μg of total RNA from WBCs and the miScript HiFlex Buffer of miScript II RT Kit (Qiagen, Hilden, Germany), enabling detection of several miRNAs and mRNAs from the same cDNA preparation.

### 2.2. Microarray Analyses and miRNA-Seq

Total RNA was extracted from WBC of 14 matched LR and 10 HR by using TRizol Reagent. 1 μg of RNA from each sample was reverse-transcribed using the High Capacity Complementary DNA reverse transcription kit (Life Technologies, Carlsbad, CA, USA) and was subsequently hybridized to a HumanHT-12 v4 Expression BeadChip kit (Illumina Inc., San Diego, CA, USA) containing 31,000 annotated genes with more than 47,000 probes and scanned using the Illumina HiScan SQ platform.

Also, miRNAs from WBCs of 6 LR and 5 HR were sequenced using Illumina’s miRNA-Seq following the standardized protocol. miRNA-Seq samples were selected randomly and adjusted for sex, gender and weight. They consisted in samples analyzed in the microarray and also additional volunteers from the Resmena study that were not included in the microarray, in order to widen the sample and validate previous results. Limma package in R was used to analyse microarray and miRNA-Seq data as published [22]. Expression data were preprocessed by background correction, log-2 transformation and quantile normalization. Corrections for multiple comparison were carried out by using the Benjamini-Hochberg procedure in both techniques. Expression microarray data are available in the ArrayExpress database (www.ebi.ac.uk/arrayexpress) under accession number E-MTAB-2604. The GEO reference GSE139837 (https://www.ncbi.nlm.nih.gov/geo/query/acc.cgi?acc=GSE139837) provides access to the miRNA-seq data.

### 2.3. Bioinformatic Study

To predict the target genes of the selected miRNAs, miRWalk 2.0 algorithms were applied. The miRWalk 2.0 is a database which links to databases such as DIANA-microT, microRNA.org, miRDB, RNA22, TargetMiner, and TargetScan, giving information of predicted and validated miRNA target sites.

### 2.4. Luciferase Reporter Constructs

Expression vectors for each miRNA were constructed by cloning the particular 3′-UTR binding region of the *GSK3B* gene provided by the bioinformatic prediction into the pmiR-GLO Dual-Luciferase miRNA Target Expression Vector (Promega, Madison, WI, USA). Primers containing *NheI* and *XbaI* restriction enzymes sites were used to amplify each specific *GSK3B* 3′-UTR binding region. PCR products were purified and subsequently digested and cloned downstream of the firefly luciferase (luc) gene after vector linearization. Primer sequences are shown in Table 1.

### 2.5. Cell Culture

Human monocytes from the leukemia cell line THP-1 (for overexpression experiments) were purchased from the ATCC (Manassas, VA, USA) and maintained in GIBCOTM RPMI-1640 Medium supplemented with 10% fetal bovine serum (FBS) and 100 U/mL penicillin-streptomycin at 37 °C in a 5% carbon dioxide humidified atmosphere. Phorbol 12-myristate 13-atectate (TPA) (Sigma-Aldrich, San Louis, MO, USA) was applied for 48 h at a final concentration of 50 ng/mL for differentiating monocytes into macrophage-like cells, and 100 ng/mL of lipopolysaccharide (LPS) (Invitrogen, Carlsbad, CA, USA) was then applied for 24 h to activate macrophages.

HEK-293T cells (for luciferase-reporter assays) were purchased from the ATCC and maintained in Dulbecco’s Modified Eagle’s Medium (DMEM) supplemented with 10% FBS and 100 U/mL penicillin-streptomycin at 37 °C in a 5% carbon dioxide humidified atmosphere.

#### mirVana miRNA Mimic Transfections

For downregulation experiments, THP-1 cells were seeded at 250,000 cells/well in 24-well plates, and differentiated into macrophage-like cells and further activated as explained in Cell Culture subsection. Activated macrophages were transiently transfected with either 20 nM or 40 nM of mirVana^®^ miR-548q mimic, mirVana^®^ miR-1185-1 mimic, or mirVana^®^ miRNA mimic negative control #1 (Applied Biosystems, Foster City, CA, USA) using 3 μL/well of Lipofectamine 2000 Transfection Reagent (Applied Biosystems). To optimize transfection efficacy, the BLOCK-iT Alexa Fluor Red Fluorescent Oligo control (Invitrogen) was transfected and fluorescence was measured 24 h post transfection (excitation 540 nm, emission 590 nm). For negative controls, 6 wells were transfected whereas 9 wells were assayed for miRNA mimics experiments.

### 2.6. Dual-Luciferase Reporter Assays

Subsequently, miRNA-target interactions were carried out in HEK-293T cells seeded at a density of 15,000 cells per well in 96-well plates. After 24 h, cells were transiently co-transfected with either 0.25 μg of empty pmiR-GLO, pmiR-GLO-548q-3′-UTR, or pmiR-GLO-1185-1-3′-UTR, and 7.5 pmol of miR-548q and miR-1185-1 mimics using 1.5 μL/well Lipofectamine 2000 (Invitrogen). Firefly luciferase activity was normalized using Renilla luciferase activity 24 h after co-transfection with a Dual-Luciferase Reporter Assay System (Promega). Determinations were carried out in three independent experiments, each assayed in triplicate.

### 2.7. Quantitative Real-Time PCR

Total RNA from THP-1 cells was extracted 24 h after transfection following the TRIzol protocol. A total of 50 ng of RNA were reverse-transcribed using miScript HiFlex Buffer of miScript II RT Kit (Qiagen). Quantitative PCR (qPCR) was performed with the CFX384 Touch Real-Time PCR Detection System (Bio-Rad, Hercules, CA, USA) using commercial Taqman probes for *GSK3B* (Applied Biosystems) or miScript Primer Assays for miRNAs (Qiagen).

Then, mRNA and miRNA expressions were calculated with the 2^−ΔΔCt^ method and normalized using glyceraldehyde-3-phosphate dehydrogenase (*GAPDH*) and small nucleolar RNA, C/D box 68 (*SNORD68*) mature miRNA as housekeeping genes, respectively.

### 2.8. Statistical Analysis

Data from humans are presented as mean ± SD and data from cells are presented as mean ± SEM. Differences between groups were calculated using two-tailed Student’s t- or ANOVA tests when indicated. In this context, *p*-values less than 0.05 were defined as statistically significant. In the case of expression microarray and Illumina’s miRNA-Seq, in order to select the best candidates to act as predictive biomarkers, a *p*-value < 0.1 was considered relevant if miRNAs were found in both platforms. Volcano figures were created by plotting the negative log of the p-value (y axis) and the mean differences between groups for each variable (x axis). An effect size (ES) of ± 1% in the expression differences and a 10xlog fold change (FC) of ± 1 in the miRNA-Seq were considered relevant. Statistical analyses and graphics were performed using GraphPad Prism version 6.0C (GraphPad Software, Inc., La Jolla, CA, USA). Pearson’s correlations were fitted to evaluate the potential correlation of miRNA expression with biochemical variables.

## 3. Results

### 3.1. Dietary Intervention

A total of 96 metabolic syndrome adults were enrolled in the RESMENA (Metabolic Syndrome Reduction in Navarra) nutritional intervention trial, where subjects followed two hypocaloric diets (−30% of energy restriction). After the dietary intervention, in order to increase the statistical power of the study, both dietary groups were merged as no differences were found either in anthropometric or in biochemical variables between groups. Aiming to identify miRNA-type biomarkers implicated in the response to the weight-loss intervention, participants were categorized into “High Responders” (HR), when weight loss was >8%, and “Low Responders” (LR) when weight loss was <8%, as previously published. In the present study, a subsample of the RESMENA cohort was used for the massive sequencing studies.

### 3.2. miR-548q and miR-1185-1 Are Overexpressed in High Responders to the Weight Loss Intervention

**Figure 1:**
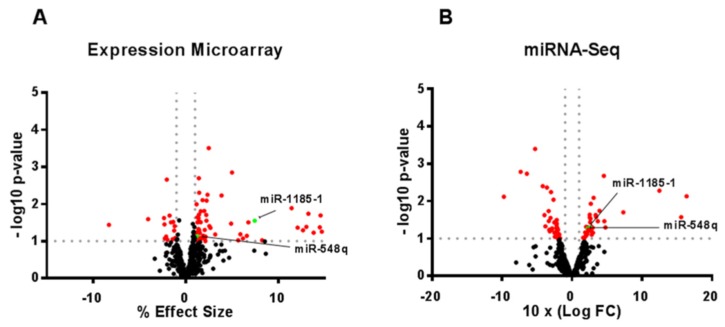
miR-548q and miR-1185-1 identification in the expression array and in the miRNA-Seq. Volcano plots of miRNAs differentially expressed between HR and LR in both the expression microarray (**A**) and in the miRNA-Seq (**B**), respectively. *p* < 0.1 and ES > 1% or 10× log FC > 1. (Expression Microarray *n* = 14 LR vs. 10 HR; miRN A-Seq *n* = 6 LR vs. 5 HR).

When combining the results from the same subjects in both platforms (expression microarray and miRNA-Seq), only two miRNAs (miR-548q and miR-1185-1) showed statistically significant differences between HR and LR subjects, with both miRNAs being overexpressed in HR (Figure 1).

**Figure 2:**
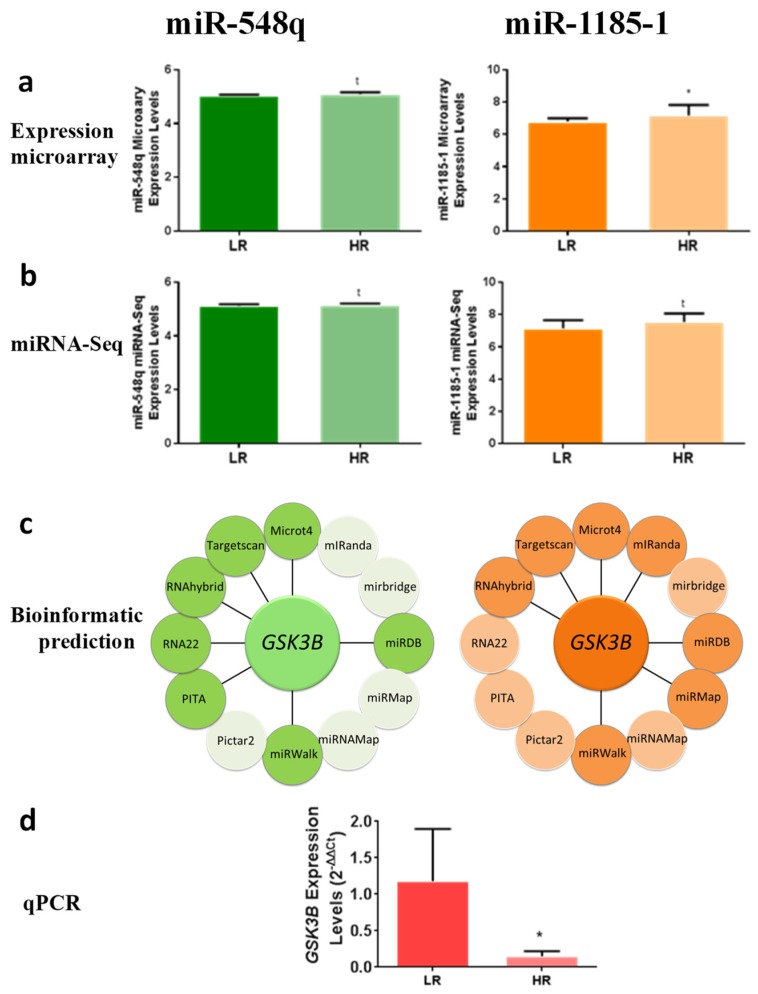
*GSK3B* is a putative target gene of miR-548q and miR-1185-1. (**a**) Microarray expression levels of miR-548q and miR-1185-1 in HR and LR to the weight loss intervention. with a line. (**b**) miRNA-Seq expression levels of miR-548q and miR-1185-1 in HR and LR to the weight loss intervention. (**c**) Bioinformatics predictions of miRWalk 2.0 for selected miRNAs. *GSK3B* appeared in 7 data bases from a total of 11. Databases which predicted *GSK3B* as a putative target gene of selected miRNAs appear connected (**d**) Validation of *GSK3B* expression profile in HR and LR WBCs by qPCR in the microarray subjects. * *p* < 0.05; *t* < 0.1 from a two-tailed Student’s t test. (Expression Microarray *n* = 14 LR vs. 10 HR; miRNA-Seq *n* = 6 LR vs. 5 HR; qPCR *n* = 14 LR vs. 10 HR).

In particular, miR-548q showed an ES = 1.29% in the expression array (*p* = 0.075), and a 10× log FC = 2.22 in the miRNA-Seq (*p* = 0.053). In contrast, miR-1185-1 showed an ES = 7.46% in the expression array (*p* = 0.028), and a 10× log FC = 2.60 in the miRNA-Seq (*p* = 0.052) as illustrated (Figure 2b,c). Interestingly, miR-1185-1 microarray expression levels were negatively correlated with serum levels of IL-6 (*r* = −0.44; *p* = 0.033; data not shown).

These differences were not significant after corrections for multiple comparisons. Nonetheless, as these miRNAs were nominally significant in both the expression array and in the miRNA-Seq, they were selected for target gene predictions with the aim of finding feasible candidates for further evaluation, considering that these experimental approaches were performed with small samples.

### 3.3. GSK3B Is a Putative Target Gene for miR-548q and miR-1185-1

**Figure 3:**
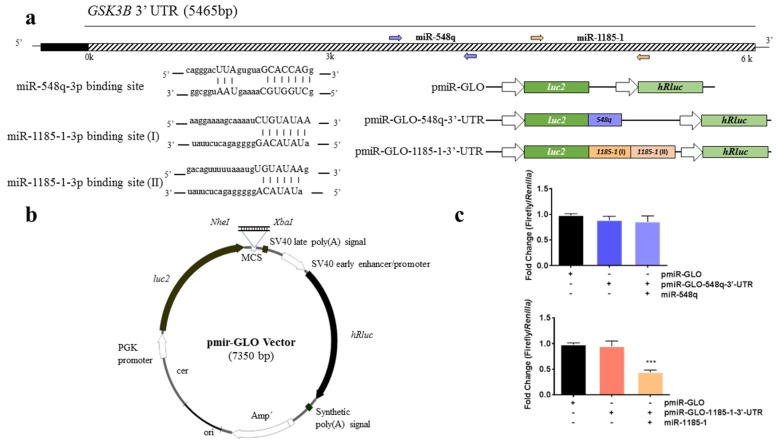
miR-1185-1 binds to the 3′-UTR of *GSK3B*. (**a**) Location of the predicted target sites for miR-548q and miR-1185-1 in the 3′-UTR of *GSK3B*. (**b**) Dual-Luciferase miRNA Target Expression Vector used to create the 3′-UTR expression vectors cloning the PCR product into the multiple cloning site (MCS). (**c**) Luciferase activity assay of pmiR-GLO-548q-3′-UTR and pmiR-GLO-1185-3′-UTR after co-transfection with either miR-548q or miR-1185-1 mimics. Normalized luciferase activity is presented as the mean ± SEM of three separate triplicate experiments. *** *p* < 0.001 from an ANOVA test.

We applied bioinformatics algorithms to screen for genes that are putative targets of the selected miRNAs. We included results from all databases linked by miRWalk 2.0 (http://zmf.umm.uni-heidelberg.de/apps/zmf/mirwalk2) and selected only those genes that appeared at least in 6 out of 11 databases. Then, the results were filtered by focusing on obesity-related genes. Finally, we noted that *GSK3B* could be a target gene for both miR-548q and miR-1185-1 (Figure 2a). According to TargetScan, the *GSK3B* mRNA 3′-UTR contains one sequence motif complementary to miR-548q and two binding sites complementary to the miR-1185-1 sequence (Figure 3a).

Interestingly, *GSK3B* mRNA levels in WBC were significantly lower (*p* = 0.014) in HR than in LR when the microarray subjects were measured by qPCR (Figure 2d), suggesting a possible regulation of these miRNAs on *GSK3B* expression.

### 3.4. miR-1185-1 Binds to the 3′-UTR of GSK3B

To determine if *GSK3B* is regulated by the binding of miR-548q and miR-1185-1 to their specific binding sites, each predicted sequence was cloned immediately downstream of the luciferase reporter gene in two different expression vectors (pmiR-GLO-548q-3′-UTR and pmiR-GLO-1185-1-3′-UTR) (Figure 3a,b). Cells co-transfected with pmiR-GLO-1185-1-3′-UTR vector and miR-1185 mimic showed lower levels of firefly/*Renilla* activity (*p* < 0.001) than controls transfected only with the pmiR-GLO-1185-1-3′-UTR vector (Figure 3c), suggesting that *GSK3B* is a target gene of miR-1185-1. However, firely/*Renilla* activity was not different between cells transfected only with the pmiR-GLO-548q-3′-UTR vector and cells co-transfected with the pmiR-GLO-548q-3′-UTR vector and the miR-548q mimic (Figure 3c).

### 3.5. miR-548q and miR-1185-1 Decrease the Endogenous GSK3B mRNA Levels

**Figure 4:**
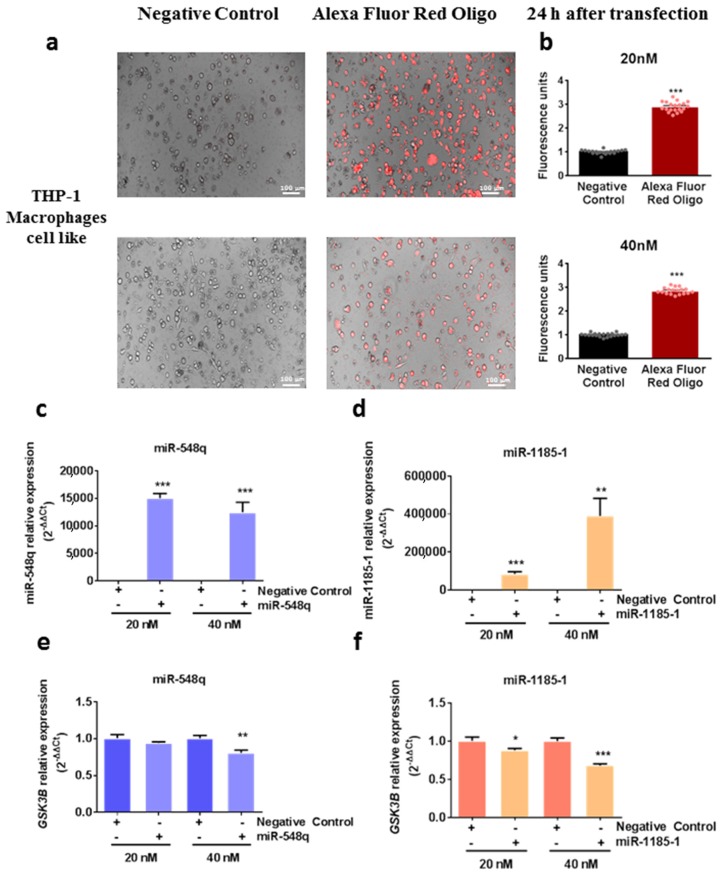
The mirVana miRNA mimic transfections in THP-1 macrophage-like cells. (**a**,**b**) Transfection optimization using the BLOCK-iT Alexa Fluor Red Fluorescent Oligo. 20 nM or 40 nM of the fluorescent oligo were transfected into THP-1 macrophage-like cells using Lipofectamine 2000 to confirm the positive transfection of small nucleotides in the cells, 24 h after transfection. Data are presented as the mean ± SEM of two different duplicate measurements divided in 5 wells. *** *p* < 0.001 from a two-tailed Student’s t test. (**c**,**d**) Efficiency of miRNA mimic transfections measuring miR-548q and miR-1185-1 levels by qPCR. (**e**,**f**) Downregulation of *GSK3B* mRNA 24h after miR-548q and miR-1185-1 mimic transfection into TPH-1 cells at different doses (20 nM and 40 nM). Data are presented as the mean ± SEM of *n* = 6 (Negative control) and *n* = 9 (mimic transfection) observations. * *p* < 0.05; ** *p* < 0.01; *** *p* < 0.001 from a two-tailed Student’s t test.

To ascertain if miR-548q and miR-1185 regulate *GSK3B* by affecting its endogenous mRNA levels, miR-548q and miR-1185-1 mimics or control were introduced into THP-1 macrophage-like cells at two different doses (20 nM and 40 nM). Next, the levels of *GSK3B* mRNA were determined by qPCR. *GSK3B* mRNA levels were reduced in miR-548q and miR-1185-1 transfected cells at the higher dose of 40 nM (*p* = 0.007 and *p* < 0.001; respectively) and in cells transfected with miR-1185-1 at a dose of 20 nM (*p* = 0.02) (Figure 4).

Altogether, these results indicate that miR-548q and miR-1185-1 are able to reduce and eventually modulate the mRNA levels of *GSK3B*.

## 4. Discussion

In the present study, we used different miRNAomic approaches and we identified two putative miRNAs that could distinguish the level of response to a specific weight loss dietary treatment. Considering that about 70% of the human genome is transcribed but only up to 2% is translated to proteins, transcriptomics has become an emerging alternative in the search for biomarkers for personalizing diagnosis, prognosis and treatment of diseases. Recent advances in the RNA-seq workflow have enabled new biomarkers to be elucidated with no prior known association with different physiological and pathological conditions [23]. Thus, next-generation sequencing (miRNA-seq) is the platform of choice for the discovery of new potential biomarkers of disease diagnosis, prognosis and therapy [24]. In this context, circulating and exosomal miRNAs have been proposed as useful in the diagnosisof diverse diseases including cancer, nervous system disorders, cardiovascular disease, diabetes and other metabolic conditions [25].

One of the best known manifestations of obesity is the alteration of adipose tissue metabolism that leads to a chronic inflammatory state characterized by the infiltration of macrophages and the secretion of proinflammatory cytokines that aggravate the process [26]. These inflammatory mediators secreted by the adipose tissue can trigger different metabolic abnormalities, impairing insulin signaling and inducing oxidative stress, leading to systemic insulin resistance and cardiovascular disease [27]. Similarly, the dysregulation of macrophage signaling can impair insulin sensitivity [28]. Likewise, hypothalamic inflammation may cause hyperphagia [29], increasing the chronic excess of nutrient intake and thus metabolic dysfunction.

Several miRNAs have been implicated in different inflammatory processes and have shown to play important roles in regulating the development and function of macrophages in the context of obesity [30]. For example, miR-21 has an essential role as a negative modulator of inflammation. In vitro overexpression studies in macrophages show that miR-21 reduces the secretion of IL-6 and increases IL-10 levels, implying an anti- inflammatory effect [31]. Another miRNA with anti-inflammatory properties is miR-24, which inhibits the production of proinflammatory cytokines in LPS-stimulated macrophages [32]. miR-124, miR-145, miR-146, miR-149, miR-155, or the miR-181 family are other miRNAs that may act as negative regulators in inflammation [33]. However, this is the first time that miR-548q and miR-1185-1 have been related to inflammation or body weight regulation.

Regarding inflammation, glycogen synthase kinase-3 (*GSK3*) has emerged as an important regulator of the process. GSK3 is a multitasking serine/threonine kinase that has over 50 substrates [34]. Initially GSK3 was thought to be only related to the metabolism of glycogen. However, interest in this kinase began growing when it was reported that GSK3 is a key member of the insulin and Wnt signaling pathways, and is involved in inflammatory responses [34]. GSK3 enhances the expression of genes activated by NF-*κ*B [35]. For example, IL-6 and MCP-1 require GSK3B (an isoform of GSK3) for efficient expression [35]. The proinflammatory role of GSK3 and the importance of its inhibitors is clear since GSK3 promotes the expression of proinflammatory cytokines such as IL-1β, IL-6 or TNF-α among others [36]. Interestingly, we found a negative correlation between microarray expression levels of miR-1185-1 and serum levels of IL-6 (*r* = −0.44; *p* = 0.033), suggesting that the regulation of *GSK3B* by miR-1185-1 may imply a reduction in inflammatory status.

On the other hand, mice receiving LPS showed decreased levels of proinflammatory cytokines, such as TNF-α, IL6, IL-1β or MCP-1, after the administration of a GSK3 inhibitor [37]. Consequently, inhibitors of GSK3 seem to provide strong therapeutic protection against inflammation and many prevalent associated diseases, such as diabetes or obesity. In obese mice, Gsk3 activity is increased in adipose tissue, and its inhibition prevents adipocyte differentiation [38,39]. In a recent study, Wang et al. demonstrated that GSK3 is essential to adipocyte differentiation and that the obesity-induced increase of *Sfrp1* expression can be reversed by GSK3 inhibitors [40], further supporting the notion that GSK3 is involved in obesity. Regarding body weight, a double knockdown of both *Gsk3a* and *Gsk3b* led to a decrease in body weight [41], whereas an overexpression of human *GSK3B* in mice skeletal muscle resulted in impaired glucose tolerance, hyperlipidemia and an increase in fat mass and body weight gain [42]. Moreover, when inhibiting Gsk3b activity, diet-induced obese mice significantly improved obesity symptoms, such as body weight gain, increased adiposity, dyslipidemia, and hepatic steatosis, due to the marked reduction of whole-body lipid content [43].

Applying different high-throughput technologies, here we found two miRNAs that interact with *GSK3B*. Our results show that both miR-548q and miR-1185-1 regulate *GSK3B* mRNA levels in a dose-dependent manner. At least for miR-1185-1, this occurred by direct binding to the associated 3′-UTR region. The regulation of miR-548q over *GSK3B* could occur in an indirect manner. Thus, it is important to highlight that miRNAs could also regulate epigenetic machinery and may be involved in an indirect epigenetic regulation. In this context, epi-miRNAs are defined as those miRNAs whose targets are, in a direct or indirect way, effectors of the epigenetic machinery. For instance, miRNAs can affect histone methyltransferases that disturb methylation of histones or DNA [44]. On the other hand, miR-548q could target the expression of some *GSK3B* enhancers or transcription factors, resulting in an ultimate reduction of *GSK3B* mRNA levels.

Although not in inflammation, *GSK3B* has been previously reported to interact with miRNAs in neoplasia and several types of cancer. In gastric tumors, miR-92, miR-182 and miR-183 expressions were increased in *Gsk3b* knockout mice [45]. In hepatocellular carcinoma, GSK3B increases miR-122 levels [46] and activates miR-181 expression [47].

To date, this is the first study to identify miR-1185-1 and miR-548q as biomarkers of weight loss that are able to predict the level of response to a dietary intervention, given their involvement in energy homeostasis. Moreover, we have demonstrated that these miRNAs are involved in the regulation of *GKS3B*, suggesting a role of these miRNAs in inflammation and body weight control. Furthermore, this result suggests that *GKS3B* could be an important mediator of weight control, and that its expression could also early differentiate between patients that are going to respond well to the dietary treatment of obesity. In summary, those patients with low expression of this gene in WBCs respond poorly to the hypocaloric dietary intervention.

This article is not devoid of limitations. First, the sample size used in both microarray and miRNA-seq was relatively low. Second, differences in miRNA expression between HR and LR were not significant after multiple comparisons, although we selected miRNAs that were differentially expressed in both techniques. Third, we cannot conclude if the changes in the expression of miR-548q and miR-1185-1 are a cause or a consequence of the obese state of the participants. Fourth, we could not conclude that the effects of the studied miRNAs over GSK3B are direct or indirect. Further studies, such as point mutations of the seed sequence, are needed to overcome this limitation. Lastly, another limitation of the present study was that, although we essayed to complete our silencing miRNA-mimic mRNA results, we were not able to measure protein levels after gene silencing. In any case, considering that it would be interesting to quantify protein levels, it is important to remark that there are different posttranscriptional regulatory mechanisms that can affect protein levels, and that a gene’s mRNA level does not often predict protein levels. Indeed, it has been reported that mRNA levels explain only around 40% of the variability in protein levels [48]. However, with the construction of expression vectors and with the transfection of miRNA-mimics instead of antimiRs, we performed two important experiments that strongly support the hypothesis that miR-548q and miR-1185-1 bind to GSK3B.

On the other hand, the study presents strengths that are important to highlight. Two different miRNAomic technologies were applied to search for differentially expressed miRNAs. Moreover, in vitro experiments complemented results from WBCs. Co-transfection experiments were performed using different expression target vectors encoding the 3′-UTR of *GSK3B* gene, with this strategy being one of the most accurate to validate the binding of miRNAs over target genes. These experiments were verified by overexpression studies with specific miRNA mimics.

The present article identified two miRNAs that are implicated in the regulation of one important inflammatory gene. miR-548q and miR-1185-1 were overexpressed in individuals that responded better to a weight loss intervention, and the mRNA expression of their target gene *GSK3B* was downregulated. This occurred through the direct binding of miR-1185-1 to the 3′-UTR of *GSK3B* and by indirect regulation of miR-548q. The fact that *GSK3B* is implicated in many inflammatory events, and considering that IL-6 levels in blood are negatively correlated with miR-1185-1, indicates that these miRNAs could have a key role in the progression of obesity-induced inflammation, and that this gene could be an important target in the battle against obesity related complications.

## Figures and Tables

**Table 1:** Primer sequences used to amplify the 3′-UTR regions of *GSK3B* and amplicon lengths.

*GSK3B*-miR-548q-F	5′-TTAGCTAGCACAGTAGGTACCGGCCTGTA-3′	668 bp
*GSK3B*-miR-548q -R	5′-TTATCTAGAGGTGGCACTCCGTGCAGT-3′	
*GSK3B*-miR-1185-1-F	5′-TTTGCTAGCCCGATGGATCACTTGGGCCT-3′	856 bp
*GSK3B*-miR-1185-1-R	5′-TTATCTAGAGGAGGTACAGCCCCACTGTT-3′	

F: Forward. R: Reverse. Underlined: *NheI* and *XbaI* target sites.
